# Safety and efficacy of tuberculosis vaccine candidates in low- and middle-income countries: a systematic review of randomised controlled clinical trials

**DOI:** 10.1186/s12879-023-08092-4

**Published:** 2023-02-24

**Authors:** Lydia Wilson, Lara Gracie, Farah Kidy, G. Neil Thomas, Krishnarajah Nirantharakumar, Sheila Greenfield, Semira Manaseki-Holland, Derek J. Ward, Tiffany E. Gooden

**Affiliations:** 1 Homerton University Hospital, Homerton Row, London, UK; 2 Institute of Medical Sciences, College of Medical and Dental Sciences, University of Birmingham, Birmingham, UK; 3 Institute of Applied Health Research, College of Medical and Dental Sciences, University of Birmingham, Birmingham, B15 2TT UK

**Keywords:** Tuberculosis, Vaccine safety, Vaccine efficacy, Infectious disease control, Clinical trials

## Abstract

**Background:**

Tuberculosis (TB) remains a leading cause of death worldwide, with 98% of cases occurring in low- and middle-income countries (LMICs). The only vaccine licenced for the prevention of TB has limited protection for adolescents, adults and vulnerable populations. A safe and effective vaccine for all populations at risk is imperative to achieve global elimination of TB. We aimed to systematically review the efficacy and safety of TB vaccine candidates in late-phase clinical trials conducted in LMICs.

**Methods:**

Medline, Embase, CENTRAL, PubMed, Clinicaltrials.gov and Greylit.org were searched in June 2021 to identify phase 2 or later clinical randomised controlled trials that report the efficacy or safety (adverse events) of TB vaccine candidates with participants of any age living in an LMIC. TB vaccine candidates listed in the 2020 WHO Global TB Report were eligible for inclusion aside from BCG revaccination. Trials were excluded if all participants had active TB at baseline. Two reviewers independently assessed papers for eligibility, and for bias and quality using the Risk of Bias 2 tool and GRADE guidelines, respectively. We report efficacy rates and frequencies of adverse events from each included trial where available and qualitatively synthesise the findings.

**Results:**

Thirteen papers representing eleven trials met our inclusion criteria. Seven vaccine candidates were reviewed across seven countries: M72/AS01, RUTI, VPM1002, H56:IC31, MTBVAC, DAR-901 and ID93 + GLA-SE. Two trials reported on efficacy: an efficacy rate of 54% (95% CI 11.5, 76.2) was reported for M72/AS01 in adults with latent TB and 3% (95% CI -13.9, 17.7) for DAR-901 in healthy adolescents. However, the latter trial was underpowered. All vaccine candidates had comparable occurrences of adverse events between treatment arms and demonstrated acceptable safety profiles; though, RUTI resulted in one serious complication in a person living with HIV. M72/AS01 was the only vaccine considered safe across a diverse group of people including people living with HIV or latent TB and healthy infants and adolescents.

**Conclusion:**

Further efficacy trials for M72/AS01 are warranted to include additional populations at risk where safety has been demonstrated. Further safety trials are needed for the remaining vaccine candidates to confirm safety in vulnerable populations.

**Supplementary Information:**

The online version contains supplementary material available at 10.1186/s12879-023-08092-4.

## Background

Globally, tuberculosis (TB) led to an estimated 1.6 million deaths in 2021 [1]. The TB burden is unevenly distributed around the world with 98% of cases occurring in low- and middle-income countries (LMICs) [2]. Of the 2 billion people affected by latent TB, around 10% will develop active TB [2]. However, the mortality rate for active TB without treatment ranges from 53 to 86% and for the first time since 2005, TB mortality increased in 2020 due to healthcare disruptions from the COVID-19 pandemic [2, 3]. TB therapies are available to some extent worldwide, however the gap between enrolment in and completion of treatment remains problematic and may be fuelling drug resistance [2]. Furthermore, the risk of TB is elevated in people living with HIV (PLWH), with TB incidence 18 times higher for PLWH than the general population [4]. PLWH with TB also have an increased risk of death, making up 14% of all TB deaths [2]. As of 2020, the World Health Organisation (WHO) End TB Strategy target of treating 40 million people with TB by 2022 achieved 50% of the target [2, 5]. Current measures are not adequate to control or eliminate this major cause of morbidity and mortality. An effective and safe vaccine for the prevention of TB, particularly for those most vulnerable such as PLWH, is imperative for attaining the global targets of reducing the harms associated with TB.

Bacille-Calmette-Guérin (BCG) is the only vaccine to be licensed for the prevention of active TB. Developed from *Mycobacterium bovis,* BCG is a live vaccine administered to more than 95% of children in high-burden countries [2]. However, disseminated BCG infection is a serious and potentially life-threatening condition caused by the vaccine that can occur in immunocompromised individuals such as PLWH not on antiretroviral therapy (ART) [2]. Given that ART initiation and adherence remains low in many LMICs, BCG is not safe for the majority of PLWH living in TB endemic areas [6, 7]. Additionally, the presence of non-tuberculous mycobacteria in the environment, coinfections (such as helminthic infections) and poor nutrition are also hypothesised to affect the immune response to BCG, and may contribute to the reduction of BCG effectiveness in many LMICs [8–10]. Even without these barriers, BCG effectiveness wanes making adolescents and adults vulnerable to subsequent infection [11].

The WHO Global Tuberculosis Report 2020 identified 14 TB vaccine candidates within the pipeline which remained unchanged in the 2021 Report [2, 4]. The most recent systematic review that investigated efficacy and safety of all TB vaccine candidates was published in 2014 and reviewed five vaccine candidates, of which two remain in the pipeline [12]. The vaccine pipeline is rapidly evolving and requires regular updates of the existing evidence. One systematic review each on the efficacy and safety of two vaccine candidates (MVA85A, M72/AS01_E_) were published in 2019 and 2020, respectively [13, 14]. Although useful, reviews focusing on individual vaccines fail to give an overview of the global progress towards the development of new vaccine candidates. Various narrative reviews have been published over the years [8, 9]; however, they perform a distinct function different than systematic reviews as they do not typically undergo systematic quality assessment. There is a need for rigorous appraisal of quality and bias to better understand any potential gaps in the evidence for any vaccine candidate within specific key populations. We therefore aim to systematically review the efficacy and safety of TB vaccine candidates within late-stage clinical trials conducted in LMICs, where a vaccine is most needed.

## Methods

This review was completed in accordance with the Cochrane Collaboration Handbook for Systematic Reviews of Interventions and was written following the PRISMA guidelines on reporting systematic reviews [15, 16].

### Eligibility criteria

We included randomised controlled trials (RCTs) in phase 2 or later clinical development with an intervention using one of the TB vaccine candidates listed within the 2020 WHO pipeline (Additional file 1). Any comparator used was considered eligible. Although BCG revaccination is included in the WHO 2020 pipeline, the safety of this approach had been recently systematically reviewed, so was not included [17]. Phase 1 trials were not considered as they typically include small samples of healthy individuals from low-risk populations; however, phase 1/2 trials were included if they met all other inclusion criteria. We considered participants of any age, sex and ethnicity in a LMIC setting reporting vaccine efficacy in prevention of TB or adverse events (AEs) as a proxy for vaccine safety. LMICs were those eligible for Official Development Assistance (ODA) as defined by the Organisation for Economic Cooperation and Development’s (OECD) and listed on the 2020 Development Assistance Committee (DAC) list [18]. We did not consider immunogenicity as an appropriate outcome for vaccine efficacy due to uncertainties regarding the immune response needed for TB protection [19, 20]. We only included safety data if it was reported as the number and proportion of events per trial participants rather than per vaccine doses. Trials were excluded if they were pre-clinical, animal studies or if all participants had active TB at baseline.

### Search strategy

Four databases (Medline, Embase, CENTRAL and PubMed) were searched in June 2021. ClinicalTrials.gov was also searched; if a registered trial had been completed without publication, the principal investigator was contacted for further information. Greylit.org was searched to identify any unpublished trials. Key words were used, including “Tuberculosis” AND “Vaccine” in conjunction with index search terms (i.e. MeSH or Emtree) where appropriate (Additional file 2). Searches were limited to human participants and RCTs if the option was available. No year of publication or language limits were set. References from included trials and existing reviews were searched to identify any additional trials potentially missed in the formal search.

### Study selection

Two reviewers (LW and LG) independently recorded the title, authors, publication date, participant demographics, setting, intervention, comparator and outcomes for each study identified from the initial search. Following removal of duplications, both reviewers independently assessed the recorded information for eligibility, reading the full text if eligibility was unclear. Any disagreements were resolved through discussion or by a third reviewer (TEG) where necessary. If two or more articles published from the same clinical trial reported data for the same outcome, then the most recent outcome data was retained.

### Data extraction

Data was extracted by one reviewer (LG) and checked for accuracy by a second reviewer (LW). Disagreements were resolved through discussion or by a third reviewer (TEG) where necessary. The Cochrane RCT Data Extraction Form [21] was used to extract all relevant data for each included trial, including details on the study characteristics, study design and participants, the number of participants randomised to each trial arm, details on the intervention and control used, how vaccines were administered, data pertaining to each relevant outcome reported for each time point and sub-group presented and results of any significant tests.

### Quality assessment and risk of bias

Quality and risk of bias was assessed independently by two reviewers (LW and LG); any disagreements were settled by discussion or by a third reviewer (TEG). Quality of each reported and relevant outcome (i.e. efficacy or AEs) was assessed using the GRADE approach, denoting evidence as high-, moderate-, low- or very low-quality [16, 22]. This review only included RCTs therefore all reported outcomes started with a high-quality GRADE assessment. Further critique either demoted the outcome’s status to lower-quality evidence or confirmed the high-quality status. Risk of bias was assessed using the Cochrane Risk of Bias 2 (RoB 2) tool for randomised trials which uses a rating system of high, low or unclear risk of bias; papers were assessed for selection, performance, detection, attrition and reporting bias [23]. Other potential biases regarding the recruitment of trial participants were also assessed [23].

### Data synthesis and analysis

It was decided a priori to not perform a meta-analysis due to heterogeneity in vaccine candidates and populations expected across clinical trials; efficacy and safety data from each trial were instead individually synthesised qualitatively by vaccine candidate. Efficacy rates were reported as per the published article or calculated by review authors. Efficacy rate is the inverse of the calculated hazard ratio and represents the ratio of advantage the intervention provides over the comparator. AEs were reported using descriptive statistics presented as numbers and percentages along with results of any statistical tests conducted by trial authors. The specific AEs reported in this review were defined as follows [24]:

- Local AE: Reactions that occur at the site of the injection; the most common are pain, redness and swelling.
- General AE: Systemic reactions that occur following vaccination including but not limited to fever, myalgia, rash and headache.
- Serious AEs (SAEs): AEs which result in death, hospitalisation or prolongation of existing hospitalisation, persistent or significant disability/incapacity or a congenital anomaly or birth defect

Some degree of local AEs may be anticipated following vaccination, and the frequency of AEs will partially depend on the vaccinated population, therefore the results reported in this review summarise differences between trial arms rather than the overall frequency of AEs.

## Results

### Results of search

**Fig. 1:**
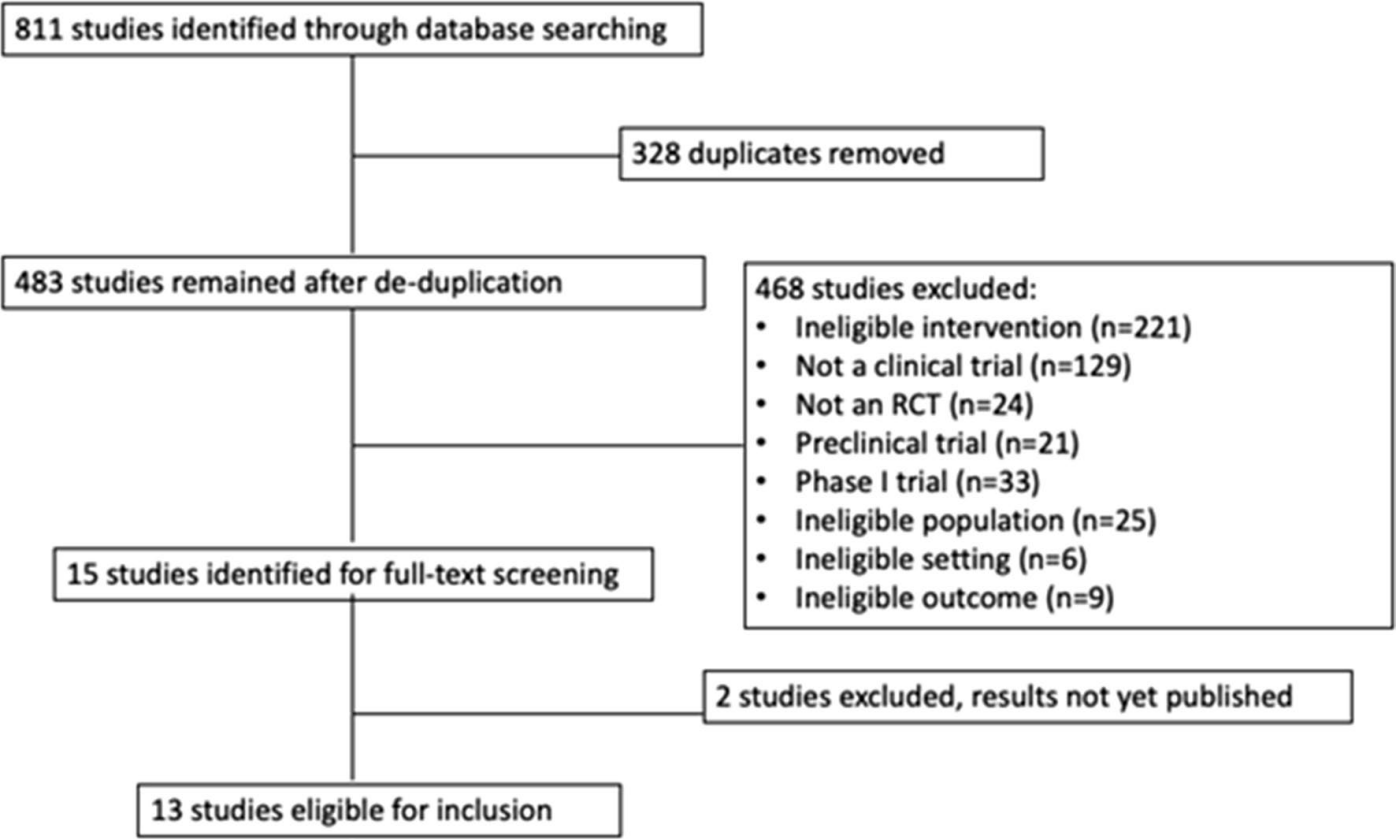
Flow chart of search results

The search generated 811 results (Fig. 1). After duplicates were removed, 483 papers remained. A further 468 papers were removed for not meeting the eligibility criteria. Fifteen papers were fully reviewed; however, two were ongoing clinical trials with no results published. The trial authors were contacted regarding any preliminary publication results or expected publication dates but they did not respond. The remaining 13 papers were included, representing 11 clinical trials. For one trial, efficacy and safety outcomes were reported in two separate papers, one at 2 years follow up [25] and another at 3 years follow up [26]. We reviewed efficacy data from the latest paper only. SAEs were reported in both papers whereas all other safety data was only reported in the earlier paper; therefore, we reviewed SAEs from the latest paper and all other safety data from the earlier publication [25, 26]. Similarly, one trial reported safety data at 12 months after vaccination in one paper [27] and in a separate paper authors reported any SAEs that occurred between 1 and 3 years after vaccination [28]. We reported data from both papers as the results did not overlap.

### Description of papers

Seven vaccine candidates were investigated in total (M72/AS01_E,B,D_, RUTI, VPM1002, H56:IC31, MTBVAC, DAR-901, and ID93 + GLA-SE) (Additional file 3). Efficacy was reported in two papers [26, 29]. All included papers reported either local AEs (n = 8), general AEs (n = 8), any AEs (n = 10), or SAEs (n = 12). All papers represented trials in phase 1/2 or 2 development, with sample sizes ranging from 48 to 3575 [25, 30]. The eleven trials were conducted across seven LMICs: The Philippines, The Gambia, South Africa, India, Kenya, Zambia and Tanzania. Of the 13 papers included, a range of age groups were represented including infants (n = 3), adolescents (n = 2) and adults (n = 9) along with vulnerable populations of PLWH (n = 3) and those with latent TB (n = 5).

### Quality and risk of bias assessment of included papers

**Fig. 2:**
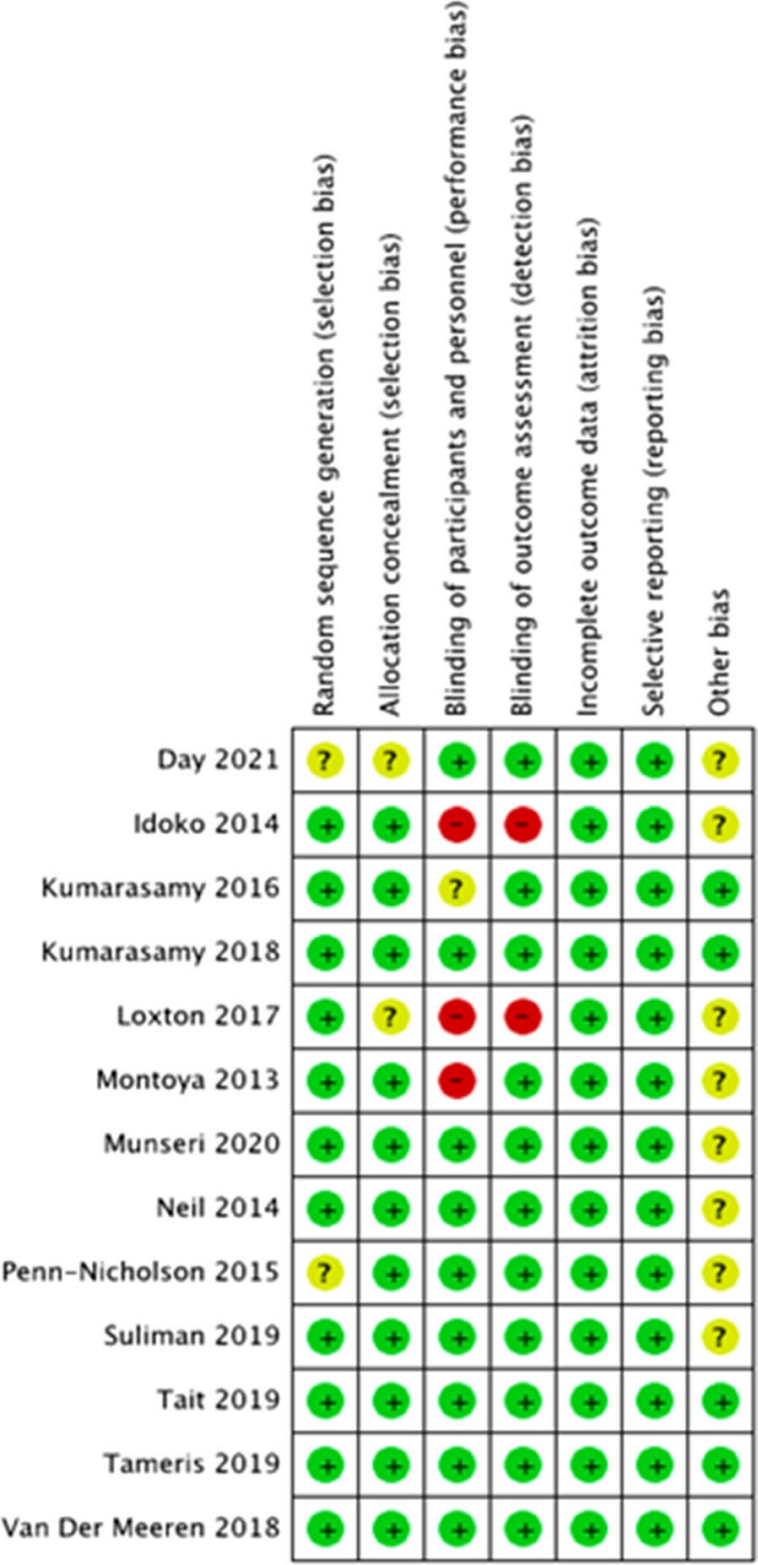
Risk of bias for each included study. Red = high risk of bias; green = low risk of bias; yellow = unclear risk of bias

Five papers were graded high quality for safety [25, 26, 29, 31, 32] (Additional file 3). Seven papers were graded moderate quality for safety due to absence of a power calculation [27, 28, 33–37]. Only one paper was graded low quality for safety as it described an open-label trial with no power calculation [30]. One paper was of high quality for efficacy [26]; however the other paper was deemed moderate quality for efficacy due to the lack of adequate power [29]. Four of the 13 papers were low risk for all bias domains [25, 26, 28, 33] and all papers were low risk of bias for attrition and reporting bias (Fig. 2). Two of the 13 papers were high risk for performance and detection bias due to being open label trials [30, 36]. One additional paper was high risk for performance bias due to unblinded staff administering the vaccine [46]. Six papers were classed as low risk of bias overall [27, 29, 31, 32, 34, 37], with a few having unclear risk of bias due to a lack of information on recruitment [29, 31, 32, 34, 37], randomisation process [30, 31] and methods of allocation [41].

### Vaccine efficacy

Tait et al. reported efficacy for M72/AS01_E_, for the prevention of active TB disease in 3575 adults with latent TB in Kenya, South Africa and Zambia [26]. Efficacy was calculated for various endpoints with PLWH excluded (unless otherwise stated). The primary endpoints were positive bacteriologic and positive PCR (polymerase chain reaction) tests before treatment initiation; both endpoints showed evidence of a significant effect (efficacy rates of 54% [95% CI 11.5, 76.2] and 64% [95% CI 19.3, 84.0], respectively) (Table 1). A sensitivity analysis of the primary endpoint included at least two positive bacteriologic tests and the efficacy rate was again supportive of effective prevention (68%, 95% CI 25.1, 86.3). The secondary endpoints were reported for participants that did not receive a test prior to treatment; thus, these endpoints were defined as bacteriologic or PCR-positive tests within four weeks after treatment initiation or a clinical diagnosis of TB. None of the these endpoints provided evidence of a significant effect: efficacy rates of 42% (95% CI -1.0, 66.5) for bacteriologic tests; 38% (95% CI -8.0, 64.7) for PCR tests; 25% (95% CI -13.8, 50.8) for clinical TB with PLWH included; and 27% (95% CI -19.8, 54.9) for clinical TB without PLWH included.

**Table 1:** Efficacy results from included papers

Efficacy Outcomes	Intervention n (%)	Control n (%)	Efficacy rate (95% confidence intervals)
Tait, 2019^a^, M72/AS01_E_	n = 1783	n = 1783	
Bacteriologically confirmed TB, sputum obtained prior to treatment initiation^b^	13 (1)	28 (2)	54.1 (11.5, 76.2)
PCR-positive TB, sputum obtained prior to treatment initiation^b^	8 (< 1)	22 (1)	64.1 (19.3, 84.0)
Bacteriologically confirmed and/or PCR-positive TB, sputum obtained up to 4 weeks after treatment initiation^b^	20 (1)	32 (2)	38.2 (− 8.0, 64.7)
Bacteriologically confirmed and/or PCR-positive TB, sputum obtained up to 4 weeks after treatment initiation	20 (1)	34 (2)	41.9 (− 1.0, 66.5)
Clinical TB	28 (2)	38 (2)	26.5 (− 19.8, 54.9)
Clinical TB^b^	27 (2)	36 (2)	25.2 (− 23.3, 54.6)
Sensitivity analysis: Two bacteriologically confirmed TB tests prior to treatment^b^	7 (< 1)	22 (1)	68.0 (25.1, 86.3)
Munseri, 2020, DAR-901	n = 314	n = 310	
New TB infection^c^	19 (6)	18 (6)	3.2 (− 13.9, 17.7)
Persistent TB infection^d^	10 (3)	5 (1)	4.4 (− 12.1, 18.5)

^a^Results from total efficacy cohort (intention to treat analysis) are presented. Per protocol analysis results were similar

^b^HIV-positive participants were excluded

^c^New TB infection was defined as conversion from IGRA-negative at baseline and at two months to IGRA-positive at any subsequent visit

^d^Persistent TB infection was defined as participants with new TB infection plus a subsequent positive IGRA at three months or later

Efficacy rates for DAR-901 were reported for the prevention of TB infection in 667 healthy adolescents in Tanzania. Two endpoints were used: new TB infection defined as IGRA (Interferon-Gamma Release Assay) positive and persistent TB infection defined as two IGRA positive tests at least three months apart. Neither endpoint provided evidence of a significant effect (efficacy rates of 3% [95% CI -13.9, 17.7] and 4% [95% CI -12.1, 18.5], respectively) (Table 1).

### Safety results

Frequencies of the most common local and general AEs and frequencies of SAEs are presented in Additional files 4, 5 and 6, respectively. All AEs and any statistical tests reported are detailed per trial in Additional file 7. Here we summarise the findings.

#### M72/AS01

In one paper reporting AEs in adults with latent TB [35], the frequency of general or any AE was comparable between intervention groups (M72/AS01_B_ at 40 μg, M72/AS01_E_ at 10 μg and 20 μg, M72/AS02_D_ at 10 μg) and control groups (M72/Saline at 40 μg and AS01_B_ alone). The only SAEs occurred in the intervention groups of M72/AS01_E_; though none were vaccine-related. In another paper that included adults with latent TB, AEs were reported more frequently in the intervention group (M72/AS01_E_) compared to the control group (sucrose) [25]. More specifically, 67% of participants in the intervention group experienced any AE whereas 45% did in the control group (relative risk 1.48, 95% CI 1.35–1.62). However, this study was not powered to detect differences in AEs between trial arms and at 3-year follow-up [26], the rate of SAEs was similar between the intervention and control groups (3% and 4%, respectively) [26]. There were two SAEs thought to be vaccine-related, one in the intervention group (pyrexia) and one in the control group (hypertensive encephalopathy).

In healthy infants outside and within the Expanded-Programme-On-Immunisation (EPI), similar rates of general or any AEs were reported between the two intervention groups (one or two doses of M72/AS01_E_) and the two control groups (meningitis vaccine for outside EPI; EPI only for within EPI) [36]. SAEs were comparable across all groups within the EPI (one SAE per group) and outside the EPI (3 SAEs each in the 1-dose intervention group and the control group; 2 SAEs from the 2-dose intervention group); none were considered vaccine-related.

In a paper reporting AEs in healthy adolescents, no SAEs were recorded in either arm though there was a difference in the rate of any AE (95% in the intervention arm of M72/AS01_E_ and 75% in the control group of saline) [37]. In adult PLWH on ART, naïve adult PLWH and adults without HIV, the rate of any AE was comparable between the intervention group (M72/AS01_E_) and their corresponding control group (saline) [27]. SAEs were only reported in PLWH (three on ART and two naïve to ART) receiving the intervention, though none were considered vaccine-related [27].

#### RUTI

Adults with latent TB with or without HIV reported comparable general AEs but reported a higher frequency of local AEs from the intervention group (RUTI of 5, 25 or 50 μg) compared to the control group (RUTI minus the MTB cells) [34]. For instance, PLWH receiving 25 μg reported more pain (n = 8, 33%), erythema (n = 11, 46%), swelling (n = 10, 42%) and induration (n = 11, 46%) compared to the control group (n = 1, 4%; n = 5, 21%; n = 4, 17%; n = 3, 13% respectively). PLWH receiving 5 μg also had higher rates of erythema (n = 9, 39%); though PLWH receiving 50 μg reported similar frequencies of AEs to the control group. Adults without HIV in the three intervention groups also experienced more pain, erythema, swelling and induration compared to adults without HIV in the control group. The rate of any AE was higher in the intervention group (100% for people living with and without HIV) than the control group (75% for PLWH, 83% for people without HIV). One vaccine-related SAE (local injection site abscess requiring hospitalisation) occurred in a participant with HIV within the intervention group and one participant with HIV in the intervention group was withdrawn due to a SAE that was not considered to be vaccine-related. No SAEs occurred in the control group.

#### VPM1002

Among healthy infants, the frequency of local AEs was similar between the intervention (VPM1002) and control groups (BCG) aside from a higher frequency of subcutaneous abscess in the control group compared to the intervention group (42% and 11%, p-value = 0.03) [30]. All infants in the intervention and control group experienced at least one AE. Two participants (6%) developed an SAE in the intervention group which was not considered vaccine-related; no SAEs occurred in the control group.

#### H56: IC31

Among adults with latent TB, the frequency of local AEs was comparable between the intervention groups (2 doses of H56:1C31 at 5, 15 or 50 μg or 3 doses at 5 μg for those without latent TB; 2 or 3 doses at 5ug for those with latent TB) and the control group (saline) [32]. An exception to this was the slightly higher frequency and rate of local pain in the 2 × 5 μg (n = 9, 60%) and 2 × 50 μg (n = 8, 53%) intervention groups compared to the control group (n = 3, 18%). There was a higher rate of fatigue, myalgia, nausea and any AE in the intervention groups; though the absolute difference was minimal as was the case for all other general AEs. Two SAEs were observed in the group receiving 2 doses of 5 μg but neither were considered vaccine related.

#### MTBVAC

In healthy adults, the frequency of any, general and local AEs was similar between the intervention (MTBVAC) and control group (BCG) [33]. Only one SAE occurred in the intervention group but it was unlikely to be vaccine-related; none occurred in the control group. In healthy infants, the absolute difference in general and local AEs were similar between the trial arms. All infants experienced at least one AE. Six infants experienced SAEs during the trial period (five from across the three intervention groups and one from the control group), though none were considered vaccine-related.

#### DAR-901

In a sample of healthy adolescents, the rate of local, general and any (38% vs 42%; p-value = 0.98) AEs were comparable between the intervention (DAR-901) and control group (saline) [29]. Six SAEs occurred in the intervention group (2%) compared to three in the control group (1%; p-value = 0.33); though none were considered vaccine-related.

##### *ID93* + *GLA-SE*

Among healthy adults, the absolute difference in local AEs were similar between the intervention groups (2 doses of 2 μg ID93 + 2 μg GLA, 10 μg ID93 + 2 μg GLA-SE or 2 μg ID93 + 5 μg GLA-SE or 3 doses of 2 μg ID93 + 5 μg GLA-SE) and the control group (saline) [31]. The only exception was those receiving 2 or 3 doses of 2 μg ID93 + 5 μg GLA-SE experienced more pain than the control group (n = 10, 71% and n = 7, 50% respectively vs n = 3, 25% in control group, p = 0.03) and more erythema than the control group (n = 4, 29% and n = 1, 7%, respectively vs n = 0, 0% in the control group, p = 0.02). The frequency of any and general AEs was comparable between the intervention and control groups. Only two SAEs occurred and both were in the control group [31].

## Discussion

Vaccination for the prevention of TB is one of the four pillars of the WHO End TB Strategy [2]. To reach the strategy’s target of an 80% reduction of TB incidence by 2030, having an effective vaccine is essential, particularly in LMIC settings and for vulnerable populations. We identified 13 papers describing 11 phase two clinical trials across seven LMICs that reported on the safety and/or efficacy of seven TB vaccine candidates (M72/AS01, RUTI, VPM1002, H56:IC31, MTBVAC, DAR-901, ID93 + GLA-SE). Of the two efficacy trials, M72/AS01 was effective at preventing active TB disease in adults with latent TB but DAR-901 was not effective in preventing TB infection in healthy adolescents. All vaccine candidates were considered safe with comparable AEs between intervention and control arms and minimal vaccine-related SAEs.

Our review highlights the lack of progression in the development of TB vaccine candidates. All trials reviewed were in phase 2 and only two papers reported efficacy [26, 29]; one of which (DAR-901) was underpowered [29]. Preclinical studies for DAR-901 indicate promising results for protection against TB disease; therefore, a sufficiently powered trial investigating efficacy for TB disease could prove useful [38]. The paper that reported efficacy for M72/ AS01_E_ was appropriately powered, considered high-quality and indicated that the vaccine was 54% protective against bacteriologically confirmed pulmonary TB disease in adults with latent TB [26]. The safety of M72/AS01_E_ was evaluated and reported in five papers comprised of adults with latent TB, infants, adolescents and PLWH. Although one paper reported a higher incidence of AEs in adults with latent TB receiving M72/AS01_E_, it was not powered to detect differences in AEs [25]; the other paper that included adults with latent TB was well-powered and reported comparable rates of AEs [35]. Based on the comparable AEs reported in all other powered trials of M72/AS01 and little evidence of vaccine-related SAEs, we conclude that M72/AS02 is a safe vaccine candidate deserving of further investigation for efficacy in additional population groups (i.e. infants, adolescents and PLWH). Our review includes two additional papers from existing reviews on M72/AS01_E_ safety though both reviews found the vaccine to be safe which correlates with our findings [14, 39].

The rate of local AEs from the RUTI vaccine in adults with latent TB with and without HIV were higher in the intervention group compared to the control group and one PLWH experienced a vaccine-related SAE in the intervention group. Whilst the safety profile was otherwise acceptable, more trials are required to further investigate these AEs in people with latent TB with and without HIV prior to advancing to large efficacy trials. The papers that reported on safety of MTBVAC, H56:IC31, ID93 + GLA-SE and DAR-901 showed comparable frequencies of AEs between the intervention and control groups with no vaccine-related SAEs, warranting further trials to investigate safety in different populations [29, 31–33]. The VPM1002 paper demonstrated an acceptable safety profile; however, the evidence was of low quality due to not using a double blind study design and not mentioning a power calculation [30]. Therefore, a more robust study design is needed to conclude the safety of this vaccine in healthy infants, among other populations.

Five of the seven countries included within our review were from the African continent; however, Asia experiences a higher TB burden [2]. Whilst a quarter of all TB cases occur in Africa, 41% occur in India, 14% in Indonesia and 12% in the Philippines [2]. Additionally, China, second to India, had one of the highest share of resistant TB infections globally in 2019 [4]. Further trials assessing efficacy and safety of TB vaccine candidates are needed in Asia to improve the generalisability of results. The wide range of vaccine efficacy demonstrated in the trials of BCG (0–90%) reinforces this point [40]. Future trials should also prioritise key populations such as PLWH and other immunocompromised individuals [41] and people with latent TB. Only three of the thirteen papers reported safety data for PLWH, one of which included sub-groups of PLWH on ART and PLWH naïve to ART [28]. Given the significant impact ART can have on the immune system, this granular analysis is essential in trials involving PLWH [42]. This is emphasised by differential frequency of AEs based on ART status [27]. None of the papers included infants diagnosed with or exposed to HIV. Mother-to-child transmission (MTCT) of HIV is a significant problem and infants with HIV are at an increased risk of TB compared to infants without HIV [43, 44]; however, with a substantial decline in MTCT due to global initiatives, a trial of exposed infants is more feasible and expected [45]. Another important group to assess safety and efficacy for is adults with latent TB for which prevalence ranges from 27 to 36% in Africa and Southeast Asia [46]. Five papers assessed efficacy and/or safety in adults with latent TB and one included adults co-infected with latent TB and HIV. Our review highlights slight variance between trial arms for the frequency of AEs in adults with latent TB, indicating the importance of their inclusion in future trials; vaccine efficacy may also differ between adults with and without latent TB.

To produce high-quality data from further clinical trials as recommend above, funding and resources for TB vaccine development will need to be constant and increased. The scientific community’s reaction to the COVID-19 pandemic and the delivery of a safe and effective vaccine proves that vaccine development can be more agile and faster than previously expected [47, 48]. An estimated 2 billion USD is needed annually for TB research and development investments [1]. Funding only reached 1 billion USD in 2021 with 121 million USD invested in new TB vaccines [49]; this is compared to the 100 billion USD that was invested to develop a COVID-19 vaccine within the first year of the pandemic [47, 48]. The WHO recognises that this level of funding is inadequate for a TB vaccine to be developed, approved and distributed in time to benefit the United Nations End TB Strategy [5].

As the first systematic review to assess efficacy and safety of TB vaccine candidates in phase two or larger trials conducted in LMICs, we provide a comprehensive, timely and important update on TB vaccine development. For instance, our review and the 2014 systematic review by Groschel et al. share only one trial in common [12]. Of the 11 vaccines in phase two or larger clinical stage listed in the WHO pipeline, we reviewed seven; the remaining four were ineligible for inclusion [2, 4]. We used Cochrane Handbook and GRADE guidelines to provide a thorough synthesis of the existing quality of evidence, enabling an improved interpretation of the collective results (a distinction from narrative reviews). Our quality and bias assessments have informed the recommendations we made for addressing gaps in the evidence. As the TB vaccine pipeline continues to evolve, it will be vital to replicate our review in the future; the data extraction, bias assessment and quality appraisal was conducted systematically and by use of reputable and accessible tools for ease of replicating. Despite the papers many strengths, there are some limitations. Publication bias was not assessed in our review. Whilst it is well documented that papers with positive findings are more likely to be accepted for publication than papers that report negative findings [50], this is less of a concern for trials due to high adherence to the pre-registration of trials on public domains. To exemplify, we identified eight eligible trials identified from clinicaltrials.gov and only two trials (completed in 2017 and 2021) had not published results at the time our search was conducted. Additionally, the TB vaccine pipeline will continue to evolve. More papers may have been published since our search (June 2021) and this review is relevant to the WHO TB vaccine pipeline presented in the 2021 Global TB Report [2, 4]. Vaccines are likely to be added to or removed from the pipeline in future reports.

In conclusion, this systematic review of late-phase clinical trials for TB vaccine candidates provides a vital update on the safety and efficacy of vaccines for people living in LMICs. This review highlights that there are multiple TB vaccine candidates with acceptable safety profiles, but most are in need of investigating safety in additional populations such as PLWH and people with latent TB before advancing to larger efficacy trials. The M72/AS01_E_ vaccine is supported by the largest body of safety data across multiple key populations and demonstrated efficacy against TB disease in a large, well-conducted trial in adults with latent TB thus a promising candidate for further research. Trialling the efficacy of M72/AS01_E_ in more vulnerable populations would be beneficial. Trialling and implementing an effective and safe vaccine would prove to be critical for achieving global control and elimination of TB; however, an increase in and constant flow of funds are required to make this a reality.

## Supplementary Information

**Additional file 1.** Eligible TB vaccine candidates. Description of all eligible TB vaccine candidates within the 2020 WHO pipeline.

**Additional file 2.** Electronic search strategy and initial hits, Search strategy for each database searched and number of hits from each.

**Additional file 3. **Study characteristics of included papers. Detailed description of each included paper including the results to the quality assessment.

**Additional file 4.** Most common solicited and unsolicited local adverse events; N (%). Frequencies of the most common local adverse events reported from each trial by each trial arm.

**Additional file 5.** Most common solicited and unsolicited general adverse events; N (%). Frequencies of the most common general adverse events reported from each trial by each trial arm.

**Additional file 6.** Any adverse events and serious adverse events; N (%). Frequencies of any adverse events and serious adverse events reported from each trial by each trial arm.

**Additional file 7. **All adverse events and serious adverse events; N (%). Frequencies of all adverse events and serious adverse events reported from each trial by each trial arm.

## Data Availability

All data generated or analysed during this study are included in this published article and its additional files.

## Abbreviations

TB: Tuberculosis
LMICs: Low- and middle-income countries
PLWH: People living with HIV
AEs: Adverse events
MDR-TB: Multidrug—resistant TB
RR-TB: Rifampicin—resistant TB
BCG: Bacille-Calmette-Guérin
ART: Antiretroviral therapy
RCTs: Randomised controlled trials
RoB 2: Risk of Bias 2
SAEs: Serious adverse events
PCR: Polymerase chain reaction
IGRA: Interferon-Gamma Release Assay
EPI: Expanded-Programme-On-Immunisation
CFU: Colony Forming Units
