# Supplementary material for: Safety and efficacy of tuberculosis vaccine candidates in low- and middle-income countries: a systematic review of randomised controlled clinical trials
Source: BMC Infect Dis. 2023 Feb 24;23:120. doi: 10.1186/s12879-023-08092-4 (PMC9951834; doi:10.1186/s12879-023-08092-4)
Supplement: Supplementary file 1 — Additional file 1. Eligible TB vaccine candidates. Description of all eligible TB vaccine candidates within the 2020 WHO pipeline. [file 12879_2023_8092_MOESM1_ESM.docx]

Additional file 1. Eligible TB vaccine candidates

| **Vaccine** | **Type** | **Strategy** | **Developer** |
| --- | --- | --- | --- |
| MTBVAC | Live | Prime | Biofabri, TBVI, Zaragosa University |
| VPM1002 | Live | Prime | SII, Max Planck, VPM |
| AEC/BC02 | Protein/Adjuvant | Prime-boost | Anhui Zhifei Longcom |
| GamTBvac | Protein/Adjuvant | Prime-boost | MoH Russian Federation |
| M72/AS01_e_ | Protein/Adjuvant | Prime-boost | GSK, Gates MRI |
| H56 + IC31 | Protein/Adjuvant | Prime-boost | SSI, Valneva, IAVI |
| ID93 + GLA-SE | Protein/Adjuvant | Prime-boost | IDRI, Wellcome Trust |
| Ad5 Ag85A | Viral vector | Prime-boost | McMaster University, CanSino |
| ChAd0x185A + MVA85A | Viral vector | Prime-boost | Oxford University |
| TB/FLU-04L | Viral vector | Prime-boost | RIBSP |
| Dar-901 booster | Whole-cell or extract | Prime-boost | Dartmouth University, GHIT |
| MIP/Immuvac | Whole-cell or extract | Prime-boost | ICMR, Cadila Pharmaceuticals |
| RUTI | Whole-cell or extract | Immunotherapeutic | Archivel Farma |

Biofabri: Institut Pasteur and Biofabri; TBVI: TuBerculosis Vaccine Initiative; SII: Serum Institute of India; VPM: Vakzine Projekt Management; MoH: Ministry of Health; GSK: GlaxoSmithKline; MRI: Medical Research Institute; SSI: Statens Serum Institute; IAVI: International AIDS Vaccine Initiative; IDRI: Infectious Disease Research Institute; RIBSP: Research Institute for Biological Safety Problems; GHIT: Global Health Innovative Technology fund; ICMR: Indian Council of Medical Research
