# Supplementary material for: Safety and efficacy of tuberculosis vaccine candidates in low- and middle-income countries: a systematic review of randomised controlled clinical trials
Source: BMC Infect Dis. 2023 Feb 24;23:120. doi: 10.1186/s12879-023-08092-4 (PMC9951834; doi:10.1186/s12879-023-08092-4)
Supplement: Supplementary file 2 — Additional file 2. Electronic search strategy and initial hits, Search strategy for each database searched and number of hits from each. [file 12879_2023_8092_MOESM2_ESM.docx]

Additional file 2. Electronic search strategy and initial hits

Medline Search:

1. Exp Tuberculosis/ OR tuberculosis.mp. (241407)

2. Vaccin$.ab. OR vaccin$.ti. (255584)

3. Exp clinical trial, phase ii/ OR exp clinical trial, phase iii/ OR exp clinical trial, phase iv/ OR exp controlled clinical trial/ OR exp randomized controlled trial/ (648089)

4. 1 AND 2 AND 3 (219) (202 limited to humans)

Embase Search:

1. tuberculosis.mp OR exp tuberculosis/ (331228)
2. vaccin*.ab OR vaccin*.ti (189736)
3. clinical trial/ OR exp controlled clinical trial/ OR exp multicenter study/ OR exp phase 2 clinical trial/ OR exp phase 3 clinical trial/ OR exp phase 4 clinical trial/ (1479160)
4. 1 AND 2 AND 3 (948)
5. limit 4 to (human and randomized controlled trial) (244)

CENTRAL search:

1. MeSH [Tuberculosis] explode (2058)
2. MeSH [Vaccines] explode (13211)
3. 1 AND 2 (198)

PubMed Search:

1. phase 2 OR phase II OR phase 3 OR phase III OR phase 4 OR phase IV (233864)

2. tuberculosis [tiab] OR tb [tiab] (Title or abstract) (240972)

3. clinical trial (1248844)

4. vaccin* [tiab] (title or abstract) (340050)

5. 1 AND 2 AND 3 AND 4 (108)

ClinicalTrials.gov:

Advance Search (62)

- Search Terms: tuberculosis AND vaccine
- Recruitment: Closed Studies
- Study Type: Interventional Studies
- Phase: Phase 2 Phase 3 Phase 4

Clinicaltrialsregister.eu:

Advance Search (7)

- Search Terms: tuberculosis AND vaccine
- Trial Phase: Phase 2; Phase 3; Phase 4
- Trial Status: Completed
