# Supplementary material for: Safety and efficacy of tuberculosis vaccine candidates in low- and middle-income countries: a systematic review of randomised controlled clinical trials
Source: BMC Infect Dis. 2023 Feb 24;23:120. doi: 10.1186/s12879-023-08092-4 (PMC9951834; doi:10.1186/s12879-023-08092-4)
Supplement: Supplementary file 3 — Additional file 3. Study characteristics of included papers. Detailed description of each included paper including the results to the quality assessment. [file 12879_2023_8092_MOESM3_ESM.docx]

Additional file 3. Study characteristics of included papers

| **Study** | **Vaccine Candidate** | **Vaccine components** | **Clinical Phase** | **Setting** | **Participants** | **Administration of vaccine** | **Comparator** | **Follow-up** | **Outcome(s)** | **Quality of evidence** |
| --- | --- | --- | --- | --- | --- | --- | --- | --- | --- | --- |
| Montoya 2013 (35) | M72/AS01_B/D/E_ | A recombinant subunit containing a fusion of two MTB antigens (Mtb32A and Mtb39A) plus the AS01 adjuvant; intended for a prime-boost strategy | 1/2 | Santa Rosa City, The Philippines | 60 adults with latent TB | 2 intramuscular doses one month apart | M72/ AS01_E_ and AS01_B_ alone | 6 months following the last vaccination | General and any adverse events, SAEs | Safety - Moderate^b^ |
| Idoko 2014 (36) | M72/AS01_E_ | A recombinant subunit containing a fusion of two MTB antigens (Mtb32A and Mtb39A) plus the AS01 adjuvant; intended for a prime-boost strategy | 2 | Fajara-Banjul, The Gambia | 297 healthy infants – 150 outside of the EPI; 147 within the EPI | 1 or 2 (1 month apart) intramuscular doses | Meningitis vaccine for outside the EPI; EPI only for within the EPI | 12 months following the last vaccination | General and any adverse events, SAEs | Safety - Moderate^a^ |
| Penn-Nicholson 2015 (37) | M72/AS01_E_ | A recombinant subunit containing a fusion of two MTB antigens (Mtb32A and Mtb39A) plus the AS01 adjuvant; intended for a prime-boost strategy | 2 | Cape Town, South Africa | 60 healthy adolescents | 2 intramuscular doses one month apart | Saline placebo | 6 months following the last vaccination | Any adverse events and SAEs | Safety - Moderate^b^ |
| Kumarasamy 2016 (27) | M72/AS01_E_ | A recombinant subunit containing a fusion of two MTB antigens (Mtb32A and Mtb39A) plus the AS01 adjuvant; intended for a prime-boost strategy | 2 | Chennai, India | 240 adults – 80 PLWH on ART; 80 PLWH ART naive; 80 people without HIV | 2 intramuscular doses one month apart | Saline placebo | 12 months following the last vaccination | Any adverse events and SAEs up to 1 year | Safety - Moderate^a, b^ |
| Kumarasamy, 2018 (28) | M72AS01_E_ | A recombinant subunit containing a fusion of two MTB antigens (Mtb32A and Mtb39A) plus the AS01 adjuvant; intended for a prime-boost strategy | 2 | Chennai, India | 214 adults – 72 PLWH on ART; 68 PLWH ART naïve; 74 people without HIV | 2 intramuscular doses one month apart | Saline placebo | 3 years after vaccination | SAEs from 1-3 years | Safety - Moderate^a, b^ |
| Van Der Meeren, 2018 (25) | M72AS01_E_ | A recombinant subunit containing a fusion of two MTB antigens (Mtb32A and Mtb39A) plus the AS01 adjuvant; intended for a prime-boost strategy | 2b | Kenya, South Africa and Zambia | 3575 adults with latent TB | 2 intramuscular doses one month apart | Sucrose placebo | 2 years after last vaccination | Local, general and any adverse events | Safety - High |
| Tait, 2019 (26) | M72AS01_E_ | A recombinant subunit containing a fusion of two MTB antigens (Mtb32A and Mtb39A) plus the AS01 adjuvant; intended for a prime-boost strategy | 2b | Kenya, South Africa and Zambia | 3575 adults with latent TB | 2 intramuscular doses one month apart | Sucrose placebo | 3 years after last vaccination | Efficacy for active TB disease and  SAEs | Safety - High Efficacy - High |
| Nell, 2014 (34) | RUTI | Made of detoxified liposomal fragmented MTB; intended for a  Immunotherapeutic strategy | 2 | Port Elizabeth, George and Bloemfontein South Africa | 95 adults with latent TB - 47 PLWH; 48 people without HIV | 2 intramuscular doses 28 days apart of either 5, 25 or 50μg | RUTI minus the detoxified fragmented MTB cells | 28 days following the last vaccination | Local, general and any adverse events, SAEs | Safety - Moderate^b, d^ |
| Loxton, 2017 (30) | VPM1002 | A recombinant Mycobacterium bovis BCG vaccine, expressing listeriolysin (Hly) from Listeria monocytogenes | 2 | Cape Town, South Africa | 48 healthy infants | 1 intradermal dose | BCG | 6 months after vaccination | Local and any adverse events, SAEs | Safety - Low^b, c^ |
| Suliman, 2019(32) | H56:IC31 | A fusion protein of three mycobacterial antigens (Ag85B, ESAT-6, and Rv266) formulated in IC31 adjuvant | 1/2a | Cape Town, South Africa | 66 adults without latent TB, 32 adults with latent TB | 2 intramuscular doses of either 5, 15 or 50μg or 3 intramuscular doses of 5μg 56 days apart | Saline placebo | 292 days after first vaccination | Local, general and any adverse events, SAEs | Safety - High |
| Tameris, 2019 (33) | MTBVAC | A live, rationally attenuated clinical strain of M. Tuberculosis with deletions in the virulence genes. It contains all the genes in BCG vaccine plus genes from Mycobacterium bovis. | 1/2 | Cape Town, South Africa | 18 healthy adults and 36 healthy infants | 1 intradermal dose of 2.5x10^3^, 2.5x10^4^ or 2.5x10^5^ CFU | BCG | 360 days after vaccination | Local, general and any adverse events, SAEs | Safety - Moderate^b,^ |
| Munseri, 2020 (29) | DAR-901 | DAR-901 is an inactivated whole cell non-tuberculous mycobacterial vaccine produced from the same Master Cell Bank used for SRL172 production. | 2b | Dar as Salaam, Tanzania | 667 healthy adolescents | 3 intradermal doses 2 months apart | Saline placebo | 3 years after first vaccination | Efficacy for TB infection,  local, general and any adverse events, SAEs | Safety –  High Efficacy - Moderate^d^ |
| Day, 2021 (31) | ID93 + GLA-SE | ID93 is a polyprotein comprised of 4 tuberculosis antigens formulated with GLA-SE adjuvant (synthetic TLR-4 agonist). | 2a | Cape Town, South Africa | 61 healthy adults | 2 intramuscular doses 56 days apart of 2μg ID93 + 2 or 5μg GLA-SE or 10μg ID93 + 5μg GLA-SE or 3 intramuscular doses 28 days apart of 2μg ID93 + 5μg GLA-SE | Saline placebo | 6 months after last vaccination | Local, general and any adverse events, SAEs | Safety - High |

SAEs: Serious adverse events; MTB: *Mycobacterial tuberculosis;* ART: Antiretroviral therapy; EPI: Expanded programme of immunisation; FCMtb: Fragmented cells of MTB

^a^ Was not a double blinded study

^b^ Either did not perform or mention a power calculation

^c^ Open label trial

^d^ Sample size smaller than power calculation
