# Supplementary material for: Safety and efficacy of tuberculosis vaccine candidates in low- and middle-income countries: a systematic review of randomised controlled clinical trials
Source: BMC Infect Dis. 2023 Feb 24;23:120. doi: 10.1186/s12879-023-08092-4 (PMC9951834; doi:10.1186/s12879-023-08092-4)
Supplement: Supplementary file 4 — Additional file 4. Most common solicited and unsolicited local adverse events; N (%). Frequencies of the most common local adverse events reported from each trial by each trial arm. [file 12879_2023_8092_MOESM4_ESM.docx]

Additional file 4. Most common solicited and unsolicited local adverse events; N (%)

| **Trial Arms** | **Local pain** | **Ulceration or erosion** | **Erythema** | **Swelling** | **Exfoliation** | **Induration** | **Scarring** | **Crusting** | **Subcutaneous abscess** | **Axillary lymphadenopathy** | **Nodules** |
| --- | --- | --- | --- | --- | --- | --- | --- | --- | --- | --- | --- |
| **Van Der Meeran, 2018, M72/AS01_E_** |  |  |  |  |  |  |  |  |  |  |  |
| Adults with LTBI intervention group (n=1786) | 613 (34) | - | 65 (4) | 191 (11) | - | - | - | - | - | - | - |
| Adults with LTBI control group (n=1787) | 74 (4) | - | 1 (<1) | 7 (<1) | - | - | - | - | - | - | - |
| Sub-cohort of the intervention group (n=148) | 121 (82)* | - | 16 (11)* | 34 (23)* | - | - | - | - | - | - | - |
| Sub-cohort of the control group (n=151) | 52 (34)* | - | 2 (1)* | 3 (2)* | - | - | - | - | - | - | - |
| **Nell, 2014, RUTI** |  |  |  |  |  |  |  |  |  |  |  |
| Adults with LTBI, HIV-, 5μg (n=12) | 9 (39) | 0 (0) | 9 (39) | 10 (43) | - | 12 (52) | - | - | 0 (0) | - | 1 (4) |
| Adults with LTBI, HIV+, 5μg (n=11) | 4 (17) | 0 (0) | 9 (39) | 5 (22) | - | 10 (43) | - | - | 0 (0) | - | 0 (0) |
| Adults with LTBI, HIV-, 25μg (n=12) | 7 (29) | 2 (8) | 12 (50) | 9 (38)) | - | 11 (46) | - | - | 1 (4) | - | 3 (13) |
| Adults with LTBI, HIV+, 25μg (n=12) | 8 (33) | 2 (8) | 11 (46) | 10 (42) | - | 11 (46) | - | - | 1 (4) | - | 9 (38) |
| Adults with LTBI, HIV-, 50μg (n=12) | 11 (46) | 2 (8) | 12 (50) | 11 (46) | - | 10 (42) | - | - | 1 (4) | - | 5 (21) |
| Adults with LTBI, HIV+, 50μg (n=12) | 9 (38) | 5 (21) | 12 (50) | 11 (46) | - | 12 (50) | - | - | 1 (4) | - | 7 (29) |
| Adults with LTBI, HIV- control group (n=12) | 5 (21) | 0 (0) | 5 (21) | 2 (8) | - | 3 (13) | - | - | 0 (0) | - | 0 (0) |
| Adults with LTBI, HIV+ control group (n=12) | 1 (4) | 0 (0) | 5 (21) | 4 (17) | - | 3 (13) | - | - | 0 () | - | 0 (0) |
| **Loxton, 2017, VPM1002** |  |  |  |  |  |  |  |  |  |  |  |
| Healthy infants intervention group (n=36) | - | 16 (44) | 32 (89) | - | - | 24 (67) | 33 (92) | 16 (44) | 4 (11) | 14 (39) | 28 (78) |
| Healthy infants control group (n=12) | - | 6 (50) | 11 (92) | - | - | 7 (58) | 11 (92) | 6 (50) | 5 (42) | 5 (42) | 8 (67) |
| **Suliman, 2019, H56:IC31** |  |  |  |  |  |  |  |  |  |  |  |
| Adults without LTBI, 2x50µg (n=15) | 8 (53)* | - | 0 (0)* | 0 (0)* | - | - | - | - | - | - | - |
| Adults without LTBI, 2x15µg (n=15) | 5 (33)* | - | 0 (0)* | 0 (0)* | - | - | - | - | - | - | - |
| Adults without LTBI, 2x5µg (n=15) | 9 (60)* | - | 1 (7)* | 1 (7)* | - | - | - | - | - | - | - |
| Adults without LTBI, 3x5µg (n=12) | 2 (17)* | - | 0 (0)* | 0 (0)* | - | - | - | - | - | - | - |
| Adults with LTBI, 2x5µg (n=12) | 5 (42)* | - | 1 (8)* | 1 (8)* | - | - | - | - | - | - | - |
| Adults with LTBI, 3x5μg (n=12) | 5 (42)* | - | 1 (8)* | 1 (8)* | - | - | - | - | - | - | - |
| Adults with LTBI control group (n=17) | 3 (18)* | - | 0 (0)* | 0 (0)* | - | - | - | - | - | - | - |
| **Tameris, 2019, MTBVAC** |  |  |  |  |  |  |  |  |  |  |  |
| Healthy adults intervention group (n=9) | - | - | 7 (78)* | 9 (100)* | 8 (89)* | - | 8 (89) | - | - | - | - |
| Healthy adults control group (n=9) | - | - | 9 (100)* | 9 (100)* | 5 (56)* | - | 9 (100) | - | - | - | - |
| Healthy infants 2.5x10^3^ CFU (n=9) | - | - | 0 (0)* | 0 (0)* | - | - | 0 (0) | - | - | - | - |
| Healthy infants 2.5x10^4^ CFU (n=9) | - | - | 0 (0)* | 0 (0)* | - | - | 0 (0) | - | - | - | - |
| Healthy infants 2.5x10^5^ CFU (n=10) | - | - | 1 (10)* | 8 (80)* | - | - | 5 (50) | - | - | - | - |
| Healthy infants control group (n=8) | - | - | 4 (50)* | 6 (75)* | - | - | 7 (88) | - | - | - | - |
| **Munseri, 2020, DAR-901** |  |  |  |  |  |  |  |  |  |  |  |
| Healthy adolescents intervention group (n=315) | 7 (3)* | 0 (0)* | - | - | - | - | - | - | - | - | - |
| Healthy adolescents control group (n=310) | 1 (<1)* | 0 (0)* | - | - | - | - | - | - | - | - | - |
| **Day, 2021, ID93 + GLA-SE** |  |  |  |  |  |  |  |  |  |  |  |
| Healthy adults 2μg ID93+2μg GLA-SE (x2) (n=15) | 5 (33)* | - | 0 (0)* | - | - | 1 (7)* | - | - | - | - | - |
| Healthy adults 10μg ID93+2μg GLA-SE (x2) (n=5) | 4 (80)* | - | 0 (0)* | - | - | 0 (0)* | - | - | - | - | - |
| Healthy adults 2μg ID93+5μg GLA-SE (x2) (n=14) | 10 (71)* | - | 4 (29)* | - | - | 3 (21)* | - | - | - | - | - |
| Healthy adults 2μg ID93+5μg GLA-SE (x3) (n=14) | 7 (50)* | - | 1 (7)* | - | - | 3 (21)* | - | - | - | - | - |
| Healthy adults control group (n=12) | 3 (25)* | - | 0 (0)* | - | - | 0 (0)* | - | - | - | - | - |

LTBI: latent TB infection; CFU: colony forming units

* Events were solicited

- Data not reported for this outcome
