# Supplementary material for: Safety and efficacy of tuberculosis vaccine candidates in low- and middle-income countries: a systematic review of randomised controlled clinical trials
Source: BMC Infect Dis. 2023 Feb 24;23:120. doi: 10.1186/s12879-023-08092-4 (PMC9951834; doi:10.1186/s12879-023-08092-4)
Supplement: Supplementary file 5 — Additional file 5. Most common solicited and unsolicited general adverse events; N (%). Frequencies of the most common general adverse events reported from each trial by each trial arm. [file 12879_2023_8092_MOESM5_ESM.docx]

Additional file 5. Most common solicited and unsolicited general adverse events; N (%)

| **Trial Arms** | **Fever** | **Arthralgia** | **Chills** | **Fatigue** | **Myalgia** | **Nausea** | **Headache** | **Malaise** |
| --- | --- | --- | --- | --- | --- | --- | --- | --- |
| **Montoya, 2013, M72/AS01_B/E/D_** |  |  |  |  |  |  |  |  |
| Adults with LTBI, M72/AS01_E_, 10μg (n=40) | 4 (10) | - | - | - | 1 (3) | - | 5 (13) | 0 (0) |
| Adults with LTBI, M72/AS02_D_, 10μg (n=40) | 4 (10) | - | - | - | 0 (0) | - | 7 (18) | 0 (0) |
| Adults with LTBI, M72/AS01_E_, 20μg (n=40) | 5 (12) | - | - | - | 0 (0) | - | 11 (28) | 2 (5) |
| Adults with LTBI, M72/AS01_B_, 40μg (n=40) | 3 (8) | - | - | - | 0 (0) | - | 7 (18) | 2 (5) |
| Adults with LTBI, Control group, M72/Saline (n=10) | 3 (30) | - | - | - | 2 (20) | - | 3 (30) | 3 (30) |
| Adults with LTBI, Control group, AS01_B_ (n=10) | 1 (10) | - | - | - | 2 (20) | - | 2 (20) | 0 (0) |
| **Idoko, 2014, M72/AS01_E_** |  |  |  |  |  |  |  |  |
| Healthy infants, within EPI intervention 1 dose (n=47) | 1 (2) | - | - | - | - | - | - | - |
| Healthy infants, within EPI intervention 2 doses (n=47) | 3 (6) | - | - | - | - | - | - | - |
| Healthy infants, within EPI control group, EPI only (n=48) | 4 (8) | - | - | - | - | - | - | - |
| Healthy infants, outside EPI intervention 1 dose (n=50) | 2 (4) | - | - | - | - | - | - | - |
| Healthy infants, outside EPI intervention 2 doses (n=50) | 0 (0) | - | - | - | - | - | - | - |
| Healthy infants, outside EPI control group, meningitis vaccine (n=50) | 0 (0) | - | - | - | - | - | - | - |
| **Van Der Meeran, 2018, M72/AS01_E_** |  |  |  |  |  |  |  |  |
| Adults with LTBI intervention group (n=1786) | 122 (7) | 30 (2) | 72 (4) | 113 (6) | 83 (5) |  | 620 (35) | 35 (2) |
| Adults with LTBI control group (n=1787) | 22 (1) | 20 (1) | 8 (<1) | 51 (3) | 24 (1) |  | 339 (19) | 5 (<1) |
| Sub-cohort of the intervention group (n=148) | 58 (39)* | - | - | 102 (69)* | 88 (60)* | - | 102 (69)* | 86 (58)* |
| Sub-cohort of the control group (n=151) | 23 (15)* | - | - | 71 (47)* | 45 (30)* | - | 70 (46)* | 40 (27)* |
| **Nell, 2014, RUTI** |  |  |  |  |  |  |  |  |
| Adults with LTBI, HIV-, 5μg (n=12) | 0 (0) | 0 (0) | 0 (0) | 1 (4) | 0 (0) | - | 2 (9) | 1 (4) |
| Adults with LTBI, HIV+, 5μg (n=11) | 0 (0) | 1 (4) | 0 (0) | 0 (0) | 0 (0) | - | 1 (4) | 1 (4) |
| Adults with LTBI, HIV-, 25μg (n=12) | 0 (0) | 0 (0) | 0 (0) | 1 (4) | 0 (0) | - | 1 (4) | 1 (4) |
| Adults with LTBI, HIV+, 25μg (n=12) | 0 (0) | 0 (0) | 0 (0) | 1 (4) | 0 (0) | - | 1 (4) | 2 (8) |
| Adults with LTBI, HIV-, 50μg (n=12) | 0 (0) | 1 (4) | 1 (4) | 1 (4) | 0 (0) | - | 5 (21) | 1 (4) |
| Adults with LTBI, HIV+, 50μg (n=12) | 0 (0) | 0 (0) | 0 (0) | 1 (4) | 1 (4) | - | 4 (17) | 1 (4) |
| Adults with LTBI, HIV- control group (n=12) | 1 (4) | 1 (4) | 0 (0) | 0 (0) | 0 (0) | - | 3 (13) | 0 (0) |
| Adults with LTBI, HIV+ control group (n=12) | 0 (0) | 1 (4) | 0 (0) | 0 (0) | 0 (0) | - | 3 (13) | 0 (0) |
| **Suliman, 2019, H56:IC31** |  |  |  |  |  |  |  |  |
| Adults without LTBI, 2x50µg (n=15) | 1 (7)* | 2 (13)* | 4 (27)* | 4 (27)* | 2 (13)* | 3 (20)* | 2 (13) | 0 (0) |
| Adults without LTBI, 2x15µg (n=15) | 1 (7)* | 1 (7)* | 1 (7)* | 3 (20)* | 5 (33)* | 2 (13)* | 1 (7) | 0 (0) |
| Adults without LTBI, 2x5µg (n=15) | 0 (0)* | 1 (7)* | 0 (0)* | 3 (20)* | 2 (13)* | 1 (7)* | 2 (13) | 1 (7) |
| Adults without LTBI, 3x5µg (n=12) | 0 (0)* | 0 (0)* | 0 (0)* | 0 (0)* | 1 (8)* | 1 (8)* | 1 (8) | 0 (0) |
| Adults with LTBI, 2x5µg (n=12) | 1 (8)* | 0 (0)* | 0 (0)* | 2 (17)* | 2 (17)* | 0 (0)* | 1 (8) | 0 (0) |
| Adults with LTBI, 3x5μg (n=12) | 0 (0) | 0 (0)* | 1 (8)* | 1 (8)* | 1 (8)* | 1 (8)* | 1 (8) | 1 (8) |
| Adults with LTBI control group (n=17) | 1 (6)* | 1 (6)* | 5 (29)* | 2 (12)* | 1 (6)* | 1 (6)* | 1 (6) | 0 (0) |
| **Tameris, 2019, MTBVAC** |  |  |  |  |  |  |  |  |
| Healthy adults intervention group (n=9) | - | - | - | 2 (22) | 2 (22)* | - | 4 (44)* | 1 (11)* |
| Healthy adults control group (n=9) | - | - | - | 3 (33) | 2 (22)* | - | 5 (56)* | 3 (33)* |
| Healthy infants 2.5x10^3^ CFU (n=9) | 0 (0)* | - | - | - | - | - | - | - |
| Healthy infants 2.5x10^4^ CFU (n=9) | 1 (11)* | - | - | - | - | - | - | - |
| Healthy infants 2.5x10^5^ CFU (n=10) | 0 (0)* | - | - | - | - | - | - | - |
| Healthy infants control group (n=8) | 1 (13)* | - | - | - | - | - | - | - |
| **Munseri, 2020, DAR-901** |  |  |  |  |  |  |  |  |
| Healthy adolescents intervention group (n=315) | 7 (3)* | - | - | - | - | - | - | - |
| Healthy adolescents control group (n=310) | 1 (<1)* | - | - | - | - | - | - | - |
| **Day, 2021, ID93 + GLA-SE** |  |  |  |  |  |  |  |  |
| Healthy adults 2μg ID93+2μg GLA-SE (x2) (n=15) | 1 (7) | 1 (7) | 0 (0) | 0 (0) | 3 (20) | 0 (0) | 0 (0)* | 1 (7) |
| Healthy adults 10μg ID93+2μg GLA-SE (x2) (n=5) | 2 (40) | 0 (0) | 1 (20) | 0 (0) | 1 (20) | 0 (0) | 0 (0)* | 0 (0) |
| Healthy adults 2μg ID93+5μg GLA-SE (x2) (n=14) | 1 (7) | 1 (7) | 2 (14) | 3 (21) | 3 (21) | 1 (70) | 0 (0)* | 0 (0) |
| Healthy adults 2μg ID93+5μg GLA-SE (x3) (n=14) | 0 (0) | 1 (7) | 0 (0) | 0 (0) | 1 (7) | 0 (0) | 1 (7)* | 0 (0) |
| Healthy adults control group (n=12) | 3 (25) | 0 (0) | 0 (0) | 0 (0) | 3 (25) | 0 (0) | 0 (0)* | 0 (0) |

CFU: colony forming units

* Events were solicited

- Data not reported for this outcome
