# Supplementary material for: Safety and efficacy of tuberculosis vaccine candidates in low- and middle-income countries: a systematic review of randomised controlled clinical trials
Source: BMC Infect Dis. 2023 Feb 24;23:120. doi: 10.1186/s12879-023-08092-4 (PMC9951834; doi:10.1186/s12879-023-08092-4)
Supplement: Supplementary file 6 — Additional file 6. Any adverse events and serious adverse events; N (%). Frequencies of any adverse events and serious adverse events reported from each trial by each trial arm. [file 12879_2023_8092_MOESM6_ESM.docx]

Additional file 6. Any adverse events and serious adverse events; N (%)

| **Trial Arms** | **Any** | **Serious** |
| --- | --- | --- |
| **Montoya, 2013, M72/ AS01_B/E/D_** |  |  |
| Adults with LTBI, M72/AS01_E_, 10μg (n=40) | 29 (73)* | 2 (5) |
| Adults with LTBI, M72/AS02_D_, 10μg (n=40) | 33 (82)* | 0 (0) |
| Adults with LTBI, M72/AS01_E_, 20μg (n=40) | 33 (83)* | 2 (5) |
| Adults with LTBI, M72/AS01_B_, 40μg (n=40) | 27 (68)* | 0 (0) |
| Adults with LTBI, Control group, M72/Saline (n=10) | 9 (90)* | 0 (0) |
| Adults with LTBI, Control group, AS01_B_ (n=10) | 7 (70)* | 0 (0) |
| **Idoko, 2014, M72/ AS01E** |  |  |
| Healthy infants, within EPI intervention 1 dose (n=47) | 14 (27)* | 1 (2) |
| Healthy infants, within EPI intervention 2 doses (n=47) | 25 (53)* | 1 (2) |
| Healthy infants, within EPI control group, EPI only (n=48) | 22 (46)* | 1 (2) |
| Healthy infants, outside EPI intervention 1 dose (n=50) | 27 (54)* | 3 (6) |
| Healthy infants, outside EPI intervention 2 doses (n=50) | 28 (56)* | 2 (4) |
| Healthy infants, outside EPI control group, meningitis vaccine (n=50) | 28 (52)* | 3 (6) |
| **Penn-Nicholson, 2015, M72/ AS01E** |  |  |
| Healthy adolescents, Intervention (n=40) | 38 (95) | 0 (0) |
| Healthy adolescents, Control (n=20) | 15 (75) | 0 (0) |
| **Kumarasamy, 2016, M72/ AS01E** |  |  |
| Adults, HIV+ ART+ intervention group (n=40) | 26 (65) | 2 (5) |
| Adults, HIV+ ART+ control group (n=40) | 27 (68) | 0 (0) |
| Adults, HIV+ ART- intervention group (n=40) | 35 (88) | 3 (8) |
| Adults, HIV+ ART- control group (n=40) | 33 (83) | 0 (0) |
| Adults, HIV- intervention group (n=40) | 13 (33) | 0 (0) |
| Adults, HIV- control group (n=40) | 13 (33) | 0 (0) |
| **Kumarasamy, 2018, M72/ AS01E** |  |  |
| Adults, HIV+ ART+ intervention group (n=40) | - | 1 (<1)^b^ |
| Adults, HIV+ ART+ control group (n=40) | - | 0 (0)^b^ |
| Adults, HIV+ ART- intervention group (n=40) | - | 0 (0)^b^ |
| Adults, HIV+ ART- control group (n=40) | - | 0 (0)^b^ |
| Adults, HIV- intervention group (n=40) | - | 0 (0)^b^ |
| Adults, HIV- control group (n=40) | - | 0 (0)^b^ |
| **Van Der Meeran, 2018, M72/ AS01E** |  |  |
| Adults with LTBI intervention group (n=1786) | 1203 (67)* | - |
| Adults with LTBI control group (n=1787) | 812 (45)* | - |
| **Tait 2019, M72/ AS01E** |  |  |
| Adults with LTBI intervention group (n=1786) | - | 51 (3)^c^ |
| Adults with LTBI control group (n=1787) | - | 64 (4)^c^ |
| **Nell, 2014, RUTI** |  |  |
| Adults with LTBI, HIV+ intervention group (n=35) | 35 (100) | 1 (<1) |
| Adults with LTBI, HIV+ control group (n=12) | 9 (75) | 0 (0) |
| Adults with LTBI, HIV- intervention group (n=36) | 36 (100) | 1 (<1) |
| Adults with LTBI, HIV- control group (n=12) | 10 (83) | 0 (0) |
| **Loxton, 2017, VPM1002** |  |  |
| Healthy infants, intervention group (n=36) | 36 (100) | 2 (6) |
| Healthy infants, control group (n=12) | 12 (100) | 0 (0) |
| **Suliman, 2019, H56:IC31** |  |  |
| Adults without LTBI, 2x50µg (n=15) | 12 (80) | 0 (0) |
| Adults without LTBI, 2x15µg (n=15) | 11 (73) | 0 (0) |
| Adults without LTBI, 2x5µg (n=15) | 12 (80) | - |
| Adults without LTBI, 3x5µg (n=12) | 6 (50) | - |
| Adults with LTBI, 2x5µg (n=12) | 8 (67) | - |
| Adults with LTBI, 3x5μg (n=12) | 8 (67) | - |
| Adults with and without latent TB, 2x5µg (n=27) | - | 2 (7) |
| Adults with and without latent TB, 3x5μg (n=24) | - | 0 (0) |
| Adults with LTBI, control group (n=17) | 9 (53) | 0 (0) |
| **Tameris, 2019, MTBVAC** |  |  |
| Healthy adults intervention group (n=9) | 9 (100) | 1 (11) |
| Healthy adults control group (n=9) | 9 (100) | 0 (0) |
| Healthy infants 2.5x10^3^ CFU (n=9) | 9 (100) | 1 (11) |
| Healthy infants 2.5x10^4^ CFU (n=9) | 9 (100) | 2 (22) |
| Healthy infants 2.5x10^5^ CFU (n=10) | 10 (100) | 2 (20) |
| Healthy infants control group (n=8) | 8 (100) | 1 (13) |
| **Munseri, 2020, DAR-901** |  |  |
| Healthy adolescents intervention group (n=315) | 126 (38) | 6 (2) |
| Healthy adolescents control group (n=310) | 142 (42) | 3 (1) |
| **Day, 2021, ID93 + GLA-SE** |  |  |
| Healthy adults 2μg ID93+2μg GLA-SE (x2) (n=15) | 12 (80) | 0 (0) |
| Healthy adults 10μg ID93+2μg GLA-SE (x2) (n=5) | 5 (100) | 0 (0) |
| Healthy adults 2μg ID93+5μg GLA-SE (x2) (n=14) | 13 (93) | 0 (0) |
| Healthy adults 2μg ID93+5μg GLA-SE (x3) (n=14) | 12 (86) | 0 (0) |
| Healthy adults control group (n=12) | 9 (75) | 2 (17) |

- Data not reported for this outcome

^a^ CFU (Colony Forming Units) – measurement of the number of viable bacteria in a sample

^b^ The SAEs reported in Kumarasamy 2018 are in addition to those reported in Kumarasamy 2016

^c^ The SAEs reported in Tait 2019 include the SAEs reported in Van der Meeren 2018

* Refers to unsolicited AEs
